# Cerebellar Transcranial Direct Current Stimulation for Motor Learning in People with Chronic Stroke: A Pilot Randomized Controlled Trial

**DOI:** 10.3390/brainsci10120982

**Published:** 2020-12-14

**Authors:** Nitika Kumari, Denise Taylor, Sharon Olsen, Usman Rashid, Nada Signal

**Affiliations:** 1 Health & Rehabilitation Research Institute, Auckland University of Technology, Auckland 1010, New Zealand; denise.taylor@aut.ac.nz (D.T.); sharon.olsen@aut.ac.nz (S.O.); usman.rashid@aut.ac.nz (U.R.); nada.signal@aut.ac.nz (N.S.); 2 Centre for Chiropractic Research, New Zealand College of Chiropractic, Auckland 1060, New Zealand

**Keywords:** transcranial direct current stimulation, cerebellum, motor learning, locomotor adaptation, split-belt treadmill, chronic stroke

## Abstract

Cerebellar transcranial direct current stimulation (ctDCS) is a non-invasive brain stimulation technique that alters neural plasticity through weak, continuous, direct currents delivered to the cerebellum. This study aimed to evaluate the feasibility of conducting a randomized controlled trial (RCT) delivering three consecutive days of ctDCS during split-belt treadmill training (SBTT) in people with chronic stroke. Using a double-blinded, parallel-group RCT design, eligible participants were randomly allocated to receive either active anodal ctDCS or sham ctDCS combined with SBTT on three consecutive days. Outcomes were assessed at one-week follow-up, using step length symmetry as a measure of motor learning and comfortable over-ground walking speed as a measure of walking capacity. The feasibility of the RCT protocol was evaluated based on recruitment, retention, protocol deviations and data completeness. The feasibility of the intervention was assessed based on safety, adherence and intervention fidelity. Of the 26 potential participants identified over four months, only four were enrolled in the study (active anodal ctDCS *n* = 1, sham ctDCS *n* = 3). Both the inclusion criteria and the fidelity of the SBTT relied upon the accurate estimation of step length asymmetry. The method used to determine the side of the step length asymmetry was unreliable and led to deviations in the protocol. The ctDCS intervention was well adhered to, safe, and delivered as per the planned protocol. Motor learning outcomes for individual participants revealed that treadmill step length symmetry remained unchanged for three participants but improved for one participant (sham ctDCS). Comfortable over-ground walking speed improved for two participants (sham ctDCS). The feasibility of the planned protocol and intervention was limited by intra-individual variability in the magnitude and side of the step length asymmetry. This limited the sample and compromised the fidelity of the SBTT intervention. To feasibly conduct a full RCT investigating the effect of ctDCS on locomotor adaptation, a reliable method of identifying and defining step length asymmetry in people with stroke is required. Future ctDCS research should either optimize the methods for SBTT delivery or utilize an alternative motor adaptation task.

## 1. Introduction

Globally, stroke is the second largest cause of disability in developing countries and the third largest cause of disability in developed countries [1]. Whilst there is some spontaneous recovery after stroke [2], and standard rehabilitation can produce additional improvements [3,4,5], over 50% of individuals are left with functional limitations at six months post-stroke [6]. One of the common limitations that persist after stroke is the inability to walk [7], with as many as 45% unable to ambulate independently in the community one year post-stroke [8]. This restricts community integration and lowers the quality of life [9].

Motor re-learning is the term used to describe the internal process by which people regain functional motor skills after stroke [10]. The underlying mechanism by which motor re-learning occurs is neural plasticity; a process in which the brain alters its structure and neural connections [11]. While motor learning often refers to the acquisition of new motor skills, a process which can take weeks, months, or even years of practice [12], this paper focuses on a type of motor learning that occurs within a shorter time-frame, known as ‘motor adaptation’. Motor adaptation is seen when perturbations are applied during an already-learnt motor task and a number of trial-and-error adjustments are made to improve task performance [13]. The adjustments occur over minutes to hours and then revert to baseline levels upon removal of the perturbation. With repeated exposure to the perturbation, learning is observed through a rapid reduction in errors [14]. Motor adaptation is particularly important during walking, where individuals may need to adjust their movements to the perturbations induced by constantly changing demands, for instance, walking on a slippery surface or uneven ground [14,15].

One rehabilitation intervention which has gained attention for its ability to promote motor adaptation is split-belt treadmill training (SBTT) [15]. Through promoting adaptive walking patterns, SBTT is able to reduce spatio-temporal walking asymmetries which accompany stroke [16]. This improved symmetry may improve walking efficiency [17,18], balance control [19,20] and may prevent secondary impairments such as musculoskeletal pain and joint degeneration [21,22]. Improving walking symmetry is considered an important determinant of stroke recovery [23,24]. Yet, unlike other features of walking, such as balance and speed, which commonly improve with rehabilitation interventions [25,26,27], only a small proportion of people experience improvements in spatial asymmetry, such as step length asymmetry [28]. This is in contrast to temporal asymmetries, such as stance time, swing time and double-limb support time, which are more responsive to standard walking rehabilitation [28] and improve with traditional treadmill interventions [29,30,31]. Furthermore, spatial asymmetry such as step length asymmetry is easy to measure in a clinical setting using visual or video observation and undergoes robust adaptation in response to SBTT [32].

During SBTT, motor adaptation, also called locomotor adaptation, is seen when one leg is placed on a faster treadmill belt and the other leg is placed on a slower belt. The intent is to induce perturbations to normal walking patterns. In response to the perturbation, initially, fast reactive feedback adjustments are made to the intra-limb spatio-temporal parameters such as stride length or stance time [32]. Within a few minutes, slow predictive feedforward adjustments are made to the inter-limb parameters such as step length, double-limb support time [32]. When healthy individuals are exposed to the perturbation, their step lengths initially become more asymmetrical. As they adapt to the uneven belt speeds, their step lengths restore to near baseline levels. When the belts are moved at equal speeds again, after-effects in the form of opposite asymmetry are seen which is de-adapted over time [32].

When SBTT is applied to people with stroke, the leg taking the shorter step length is placed on the faster belt [33]. Step length is defined by the distance between the two feet at heel strike of the leading leg [34]. According to literature, most people with stroke have a shorter step length on the less affected leg [35,36,37,38]. This may arise from the more affected leg being weaker and less able to maintain a single stance when the less affected leg is in the swing phase. Placing the leg with the shorter step length on the faster belt augments the error associated with the asymmetrical walking pattern [33,39]. This is thought to make the person aware of the asymmetry, which was previously perceived as normal, so that it can be corrected. Exposure to SBTT initially worsens asymmetry, but individuals with some capacity in the cerebello-cortical pathways will start making adjustments to increase the step length of both legs, particularly of the leg on the faster belt [40]. This adaptation occurs within 10–15 min [32]. When the belt speeds are returned to normal, the adapted walking pattern is maintained, which results in an after-effect of more symmetrical step lengths, indicating storage of the adapted pattern [14]. While most people with stroke demonstrate the ability to adapt to SBTT [41], the adaptation occurs more slowly than in healthy individuals [42,43]. Nevertheless, just a single session of SBTT in people with chronic stroke can result in short-term improvements in step length symmetry [41,44]. These single session effects are partially carried over to over-ground walking [40], but with repeated sessions, there are sustained improvements in step length symmetry during over-ground walking [16,45]. This is particularly significant for the stroke population, who often show immediate improvements in performance with training, but fail to retain improvements [10].

A large body of research has been devoted to investigating methods to harness neural plasticity after stroke, and this has included the exploration of non-invasive brain stimulation techniques as adjuncts to standard rehabilitation interventions [46,47]. One such intervention is transcranial direct current stimulation (tDCS), a non-invasive brain stimulation technique that modulates neural plasticity via a continuous weak electric current delivered to the scalp through positively or negatively charged electrodes [48]. tDCS is known to influence neural cell membrane potential and alter the synaptic function of various receptors, synapses and neurotransmitters [48,49]. In people with stroke, the lesioned or non-lesioned primary motor cortices (M1) are commonly targeted with tDCS [50]; however, lack of change in kinetics, kinematics and M1 cortical activity of lower extremities [51] warrants targeting other brain structures with tDCS. Recently the cerebellum has been targeted due to its involvement in error-based motor adaptation [52,53]. In people with cortical lesions, where the cerebellar networks are intact, but their influence on the primary motor cortex is impaired, tDCS can be applied over the cerebellum (ctDCS) to modulate its excitability and influence its control over the cortex [54]. When ctDCS is applied during SBTT or other motor adaptation task, it can potentially facilitate the motor adaptation process as the individual adjusts their movement patterns to the perturbation [34].

Previous research investigating ctDCS has primarily investigated the efficacy of single session applications during motor skill and motor adaptation tasks in healthy individuals (see review by van Dun et al. 2016 [55]). A 15–20 min session of anodal ctDCS (current density of 0.08 mA/cm^2^) delivered ipsilateral to the training limb in healthy individuals has the potential to improve motor performance beyond the training period [56]. Similarly, repeated application of anodal ctDCS during task training over three consecutive days can enhance motor skill learning [57] and prolong the maintenance of learnt walking patterns in healthy individuals [58]. There is limited research concerning the effects of ctDCS in people with stroke [59]. One study demonstrated that a single session of contra-lesional anodal ctDCS delivered during task training enhanced performance of a balance adaptation task as measured by a post-intervention improvement in tandem standing balance [59]. While the few studies that have looked at the effects of single session ctDCS are promising, no studies have investigated the efficacy of repeated application of ctDCS in people with stroke. As rehabilitation interventions are commonly given over multiple sessions, it is important to evaluate the effect of repeated sessions of ctDCS during SBTT on measures of walking symmetry. Prior to conducting a fully-powered RCT, a pilot RCT was conducted to establish feasibility of the study protocol and the ctDCS intervention delivered in conjunction with SBTT. The feasibility of the RCT protocol was investigated in terms of recruitment, retention, protocol deviations and data completeness. The feasibility of anodal ctDCS + SBTT intervention was assessed in relation to adherence, intervention fidelity and safety.

## 2. Materials and Methods

### 2.1. Study Design and Setting

This was a double-blinded, parallel-group, sham-controlled, pilot RCT. Participants were randomly allocated to one of two intervention groups: active anodal ctDCS or sham ctDCS. Participants were blinded to group allocation but were aware that they would be randomized to one of the two conditions where the stimulation intensity differed. The principal investigator, who applied the ctDCS and performed outcome measurements, was blinded to group allocation. Blinding to group allocation was ensured by using two separate battery-operated constant current stimulators (HDCstim part of HDC kit, Magstim) which were labelled with two separate codes; these had been labeled and pre-programmed as either active or sham by another researcher. Blinding was maintained until data processing and analysis were complete. Study outcomes were collected immediately before the intervention, immediately after the intervention, and at follow-up one week later.

The study was undertaken at a movement analysis laboratory at Auckland University of Technology (Auckland, New Zealand). The study was approved by the New Zealand Health and Disability Ethics Committees (17/STH/147), Auckland University of Technology Ethics Committee (18/7), and Waitemata District Health Board (RM13997). The experimental protocol was registered with the Australian New Zealand Clinical Trials Registry (ACTRN12618000094279). All participants provided written informed consent before data collection commenced.

### 2.2. Participants

Participants were included if they were aged 18 years or over, had sustained a single, unilateral stroke more than six months ago and had some difficulty in walking. Exclusion criteria included the inability to continuously walk for five minutes, radiological or clinical evidence of a cerebellar lesion, the affected leg having a shorter step length, history of orthopedic, cardiac, or neurological conditions that could interfere with walking and any contraindications to the application of ctDCS [60].

**Figure 1:**
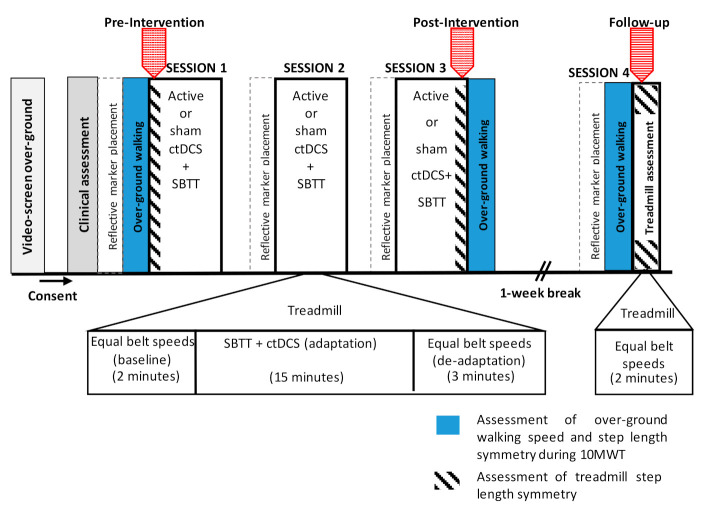
An illustration of the study protocol.

Participants were recruited through local private rehabilitation providers, local hospitals, stroke advocacy networks and professional networks. All individuals who expressed an interest in the study were provided with a participant information sheet. Potential participants were initially screened over the telephone by a trained researcher. Potential participants were then offered a face-to-face appointment at the laboratory to confirm which leg had the shorter and longer step length. This was carried out using video observation of the participant as they walked with their usual walking aid on a 20-meter walking track (refer to video-screening in Figure 1). Visual analysis of step length asymmetry has been used in previous research [40] and was chosen over other measurement methods, such as an electronic pressure sensitive walkway [45,61,62] and 3D motion analysis [44], to reduce the time-burden for participants [44,63,64] and increase feasibility within a clinical setting. A decision to include only individuals who had a longer step length on their affected leg allowed us to compare our results with those of Jayaram, Tang, Pallegadda, Vasudevan, Celnik and Bastian [34].

### 2.3. Randomisation

Following consent, participants were allocated via pseudo-randomization to either the active ctDCS or sham ctDCS group using the Minim program [65]. Minimization with a priori prognostic factors for response to treatment intervention (age and comfortable walking speed) was used. This method lowers the risk of unmatched groups when the sample size is small [66] and is considered methodologically equivalent to true randomization [67].

### 2.4. Study Procedures

Each participant attended four sessions; three intervention sessions held on consecutive days and a follow-up assessment session one week later (Figure 1). Following screening and consent, a clinical assessment was performed to collect demographic and medical information and assess stroke severity using the National Institute of Health Stroke Scale (NIHSS) [68] and global disability using the simplified modified Rankin Scale (SMRS) [69]. Next, tests of over-ground walking speed and over-ground step length symmetry during the timed 10-meter walk test (10MWT) (represented by blue in Figure 1) were performed as pre-intervention measures. These measures were repeated at the end of session 3 (post-intervention) and at session 4 (follow-up).

During the three intervention sessions (sessions 1–3), participants walked on a split-belt treadmill for 20 min; this included two-minutes of walking with equal belt speeds (baseline phase), 15 min of SBTT during which the belt speeds were uneven and participants received either active ctDCS or sham ctDCS (adaptation phase), and a further three-minutes of walking with equal belt speeds (de-adaptation phase) (refer to Figure 1). The three-minute de-adaptation phase was included to ensure a safe transition from the split-belt treadmill to over-ground walking [70]. Measures of treadmill step length symmetry were collected during the intervention sessions, in the baseline phase of session 1 (pre-intervention) and the first five strides of the de-adaptation phase of session 3 (post-intervention). Treadmill step length symmetry was reassessed one week later in session 4 (follow-up), during two-minutes of treadmill walking at equal belt speeds (without ctDCS). Sessions were held at the same time of day and lasted approximately two hours.

### 2.5. Data Collection and Data Processing

At each of the four data collection sessions, 33 reflective markers were attached to the participant with double-sided tape, according to the Cleveland clinic model [71]. Participants performed over-ground walking while the marker position data were collected using an eight-camera three-dimensional (3D) motion capture system (Vicon Nexus 2.4, Vicon Motion Systems Ltd., Los Angeles, Culver City, CA, USA). During treadmill walking, along with marker position data, force data were collected from the force plates embedded in split-belt treadmill belts. Kinematic data were sampled at 200 Hz and kinetic data at 1000 Hz. Participants initially walked over-ground for 10-meters (10MWT). Three trials were performed with their usual walking aids. Participants then completed the treadmill training task. Participants wore a safety harness and were positioned in the middle of the treadmill. They were instructed to look straight ahead while walking on the treadmill and hold onto a front handrail adjusted to elbow-height. Data were collected during the last minute of the baseline phase, and throughout the adaptation and de-adaptation phases, excluding rest periods.

The raw over-ground and treadmill kinematic and force data were processed and gap-filled in Vicon software. The data were further processed with custom-made MATLAB software [72], which low-pass filtered the force data at 10 Hz and identified heel-strike and toe-off events. Heel-strike and toe-off events were visually checked for accuracy. A modified version of step length was calculated as the anteroposterior distance between the lateral malleoli reflective markers of each leg at heel strike of the leading leg [34]. Step length data were used to calculate step length symmetry values (see the section on Outcome Measures).

### 2.6. Interventions

#### 2.6.1. Split-Belt Treadmill Training

**Figure 2:**
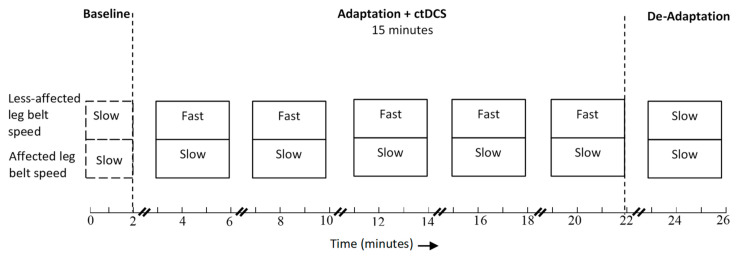
Split-belt treadmill training protocol.

Both active and sham groups walked on a split-belt treadmill (Bertec Corporation, Columbus, OH, USA), which comprised two separate belts capable of moving together or at different speeds. The speed of the belts during each of the three phases (baseline, adaptation, de-adaptation) was determined based on the participant’s comfortable over-ground walking speed. The individual’s comfortable walking speed was chosen to set the belt speeds as it has been found to be associated with larger after-effects in healthy individuals [73]. The slow belt speed was set to 80% and the fast belt speed to 160% of comfortable walking speed, so as to move the belts at a speed ratio of 1:2 [16,45]. The belt speeds were changed abruptly with an acceleration of 0.05 m/s^2^. When both belts were set to the same speed, the speed was set to 80% of the comfortable walking speed as people with stroke walk slower on the treadmill [74]. During the *baseline phase*, the participant walked on the treadmill with both belts moving together at a slow speed for two minutes. During the *adaptation phase,* the speed of the belt under the leg with the shorter step length was increased to the fast speed [41,45], while the belt under the leg with the longer step length was kept at the slow speed. This exaggerates the individual’s step length asymmetry [39]. Mandatory sitting or standing breaks were given every three-minutes to prevent fatigue. ctDCS was delivered only during the *adaptation phase* and was switched off during rest periods. After five bouts of adaptation, both belts were returned to slow speed and the participant walked for a further three minutes. This is referred to as the *de-adaptation phase*. Figure 2 illustrates the split-belt treadmill protocol.

#### 2.6.2. ctDCS

Active anodal or sham ctDCS was delivered via a pair of rubber electrodes (5cm × 5cm) encased in saline (0.9%) soaked sponges. The anode was positioned 3 cm lateral to the inion [34,53] towards the affected side to target the lateral cerebellar hemisphere contralateral to the lesioned side (contra-lesional stimulation) [59]. The 3 cm lateral to the inion electrode placement has shown to modulate cerebellar excitability during SBTT reported as a reduction in cerebellar brain inhibition with TMS-induced motor evoked potentials [53]. The contra-lesional cerebellum was stimulated because this has crossed connections with the ipsi-lesional primary motor cortex, so enhancing its function might strengthen the cerebellar-cortical connections to the affected hemisphere [75]. The cathode was placed over the ipsilateral buccinator muscle [34]. In the active anodal ctDCS group, a constant current of 2 mA was delivered during the adaptation phase of SBTT to produce a current density of 0.08 mA/cm^2^ [55]. This current intensity and density have been shown to be effective and safe [56,76]. The stimulation was delivered over five 3 min bouts, which included 30 s ramp up and ramp down, resulting in a total stimulation duration of 10 min. In the sham ctDCS group, a constant current of 2 mA was delivered for 30 s and then turned off automatically during each of the five bouts of SBTT [34].

### 2.7. Outcome Measures

#### 2.7.1. Primary Measure

The primary outcome of the study was motor learning, which was evaluated based on pre-intervention to follow-up change in step length symmetry during both over-ground and treadmill walking. Step length symmetry is an inter-limb kinematic variable that undergoes robust adaptation in response to split-belt treadmill walking [32]. Step length symmetry was calculated separately for the treadmill and over-ground walking. This was done to ensure the results could be interpreted based on their minimal detectable change (MDC). Pre-intervention and follow-up assessment utilized the average of all the strides of the treadmill (baseline phase) or over-ground walking at session 1 and follow-up, respectively.

##### Treadmill Step Length Symmetry

Treadmill step length symmetry was calculated using the symmetry index, as per the following equation [77]:

$$Symmetry\;index=\;\frac{Affected\;leg\;step\;length\;\left(A\right)}{Affected\;leg\;step\;length\;\left(A\right)+less\;affected\;leg\;step\;length\;\left(LA\right)}$$

A symmetry index of 0.50 indicates perfect step length symmetry. Values >0.5 indicate the affected leg took the longer step. Values <0.5 indicate that the less-affected leg took the longer step leg [77].

##### Over-Ground Step Length Symmetry

Over-ground step length symmetry was calculated using the symmetry ratio, as per the following equation [78,79]:

$$Symmetry\;ratio=\;\frac{Longer\;step\;length\;\left(L\right)}{Shorter\;step\;length\;\left(S\right)}$$

This equation produces symmetry ratios of 1 and over, with a value of 1 indicating perfect step length symmetry.

#### 2.7.2. Secondary Measures

##### Pre- to Post-Intervention Change in Step Length Symmetry

Pre-intervention to post-intervention change in step length symmetry during treadmill and over-ground walking represented the magnitude of after-effects and magnitude of carry-over, respectively. The post-intervention assessment constituted the average of the first five strides of the session 3 treadmill de-adaptation phase [41] and first over-ground walking trial [40].

##### Comfortable Over-Ground Walking Speed

Comfortable over-ground walking speed was recorded as a measure of walking capacity. This was evaluated with the timed 10MWT based on the average of three trials [80]. The 10MWT is a reliable and valid measure of walking performance in people with stroke [80,81]. This was measured pre-intervention (session 1), after the three intervention sessions (post-intervention session 3) and one week later (session 4).

### 2.8. Feasibility Measures

The feasibility of the RCT protocol and the intervention (anodal ctDCS during SBTT) were evaluated in relation to pre-determined criteria outlined in Table 1. The data related to these criteria were recorded on data collection sheets.

### 2.9. Data Analysis

Change-scores for both treadmill and over-ground step length symmetry were calculated as pre- minus post-intervention values such that a positive value would indicate improvement. Motor learning and walking ability measures were analyzed using descriptive statistics (means and mean differences). The feasibility issues were evaluated through percentage where applicable.

## 3. Results

### 3.1. Feasibility of the Research Protocol

#### 3.1.1. Recruitment

**Figure 3:**
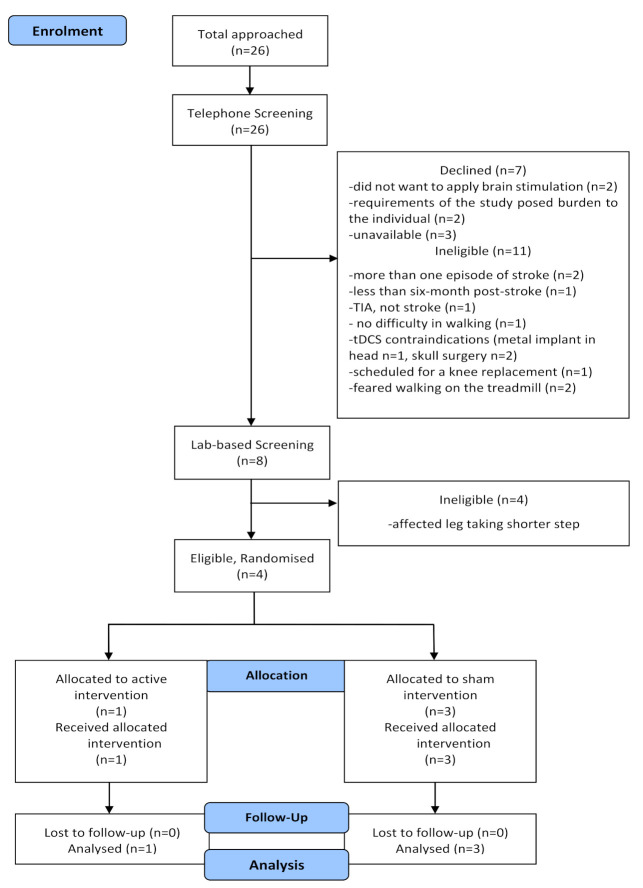
CONSORT study flow diagram.

Recruitment was undertaken from February 2018 until May 2018. Over the four-month recruitment period, 26 individuals expressed interest in participating in the study. At telephone screening, seven declined to go ahead with further screening and 11 were deemed ineligible as they did not meet the inclusion criteria (Figure 3). The remaining eight individuals were offered an appointment for lab-based screening, of which four individuals were excluded as their step length was shorter on the affected side. Four participants were recruited into the study. Refer to Figure 3 for an outline of the study flow.

#### 3.1.2. Characteristics of the Sample

Participants’ demographics and stroke characteristics are presented in Table 2. The participants were between the age of 51 to 80 years and had moderate to significant walking disability based on their over-ground walking speed [82]. Stroke severity was mild, according to NIHSS and SMRS scores [68,69]. 

#### 3.1.3. Retention

All four participants completed the full study protocol.

#### 3.1.4. Protocol Deviations

During the lab-based screening, high intra-individual variability in step length symmetry posed a challenge in determining the side of asymmetry during over-ground walking. To supplement the video assessment of over-ground walking, an additional video assessment of treadmill walking was undertaken.

#### 3.1.5. Data Completeness

Data completeness of 100% was achieved.

### 3.2. Feasibility of the Intervention

#### 3.2.1. Adherence

Participants completed all three intervention sessions.

#### 3.2.2. Intervention fidelity

##### ctDCS

All participants received their allocated ctDCS intervention according to the planned location and intensity. The ctDCS interventions lasted an average of 26 min (range 19 to 38 min); this included 10 min of stimulation delivery (five 2-min bouts) and the remaining minutes were spent resting. Overall, 96.7% of the stimulation bouts were delivered correctly, with only two occasions where ctDCS was delivered during rest periods due to a technical fault.

##### Split-Belt Treadmill Training

The speed of the fast and slow belts was set at the desired ratio for all sessions. The protocol dictated that the leg with the shorter step length be placed on the fast belt. Due to difficulty in determining which side had the shorter step length using video observation of over-ground walking, this was assessed using video observation of treadmill walking. However, on retrospective comparison of the baseline video recording of treadmill walking with the baseline 3D motion analysis of treadmill walking, there was a discrepancy in three of the four participants. Based on the video observation of treadmill walking, the less-affected leg was deemed to have the shorter step length, and, therefore, it was allocated to the fast belt. However, according to the pattern of walking observed on the treadmill via 3D motion analysis, the affected leg should have been placed on the fast belt as it had the shorter step length. This discrepancy occurred for three out of four participants (75%). Determination of step length in 3D motion analysis system as ankle-to-ankle instead of heel-to-heel may have led to the discrepancy. Refer to Table 3 for the comparison of step length asymmetry direction determined during video observation (over-ground walking) and 3D motion analysis data (treadmill walking) with symmetry threshold determined by the step length difference. The duration of SBTT at each phase was as per the planned protocol.

#### 3.2.3. Intervention Safety:

There were no adverse events. The participants who received sham ctDCS reported no sensation in 66.6% of sessions, twitching of the cheek in 33.3% of sessions, and a metallic taste in 22.2% of sessions. The participant who received active anodal ctDCS reported tingling on the cheek in all sessions. The participants perceived all the sensations as mild and related to ctDCS.

### 3.3. Outcome Measures

**Figure 4:**
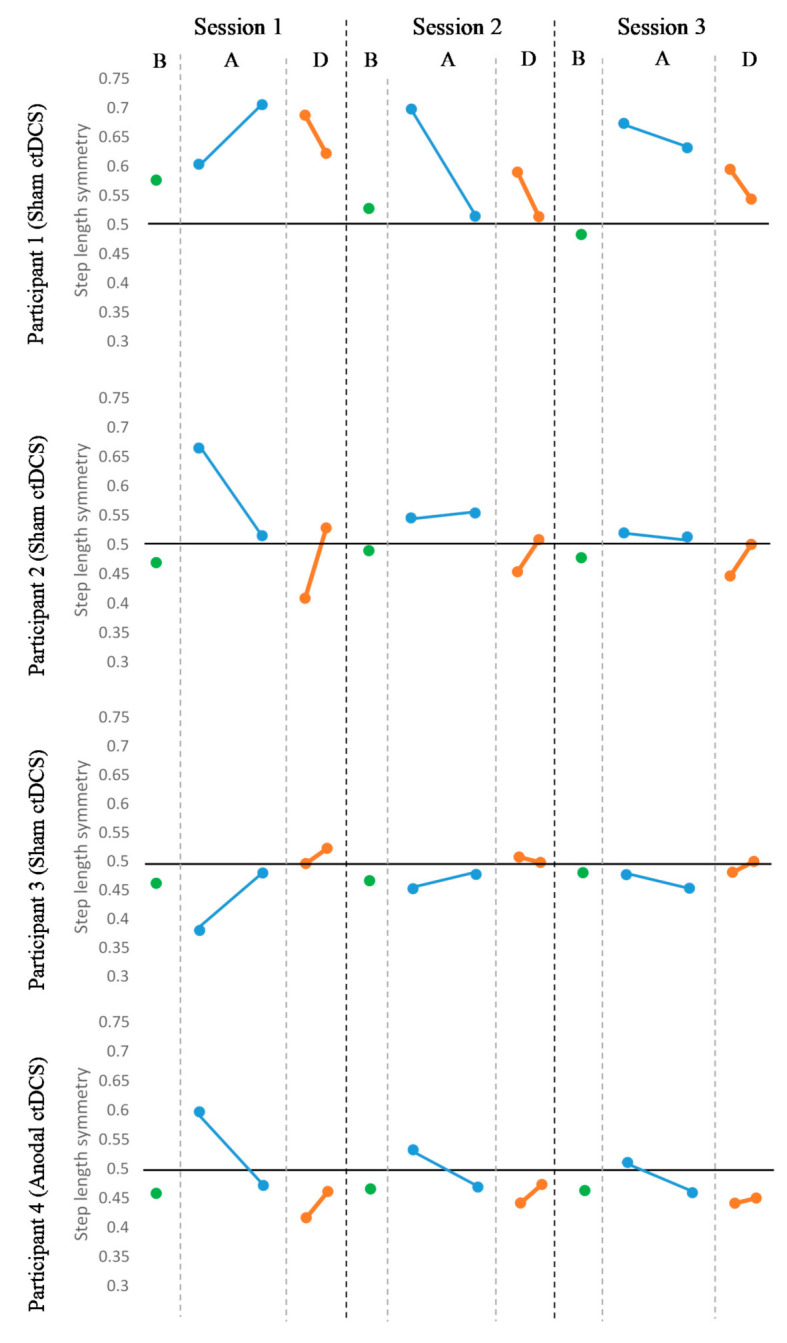
Treadmill step length symmetry over three consecutive sessions. The black horizontal line represents perfect symmetry. B: mean step length symmetry at the baseline phase (green), A: mean step length symmetry of first five and last five strides of the adaptation phase (blue), D: mean step length symmetry of first five and last five strides of the de-adaptation phase (orange).

As only four participants completed the experimental protocol, individual data are presented rather than group data. Individual changes in symmetry were interpreted based on MDC reported in the literature, which was 0.068 for treadmill step length symmetry [77] and 0.15 for over-ground step length symmetry [78]. All the participants demonstrated variable patterns of treadmill step length symmetry during the three consecutive intervention sessions. For participant 1 (sham ctDCS) and participant 3 (sham ctDCS), their treadmill step lengths became more asymmetrical at the end of the adaptation phase in sessions 1 and 3, indicating they did not adapt to the split-belt treadmill walking. Their de-adaptation phase step length symmetry was variable. The trends for baseline, adaptation, and de-adaptation phase for participant 2 (sham ctDCS) and participant 4 (anodal ctDCS) resembled the expected pattern except that their initial symmetry at the start of the adaptation phase was exaggerated in the opposite direction to the baseline symmetry (Figure 4).

#### 3.3.1. Treadmill Step Length Symmetry

##### Pre-to Post-Intervention Assessment

**Figure 5:**
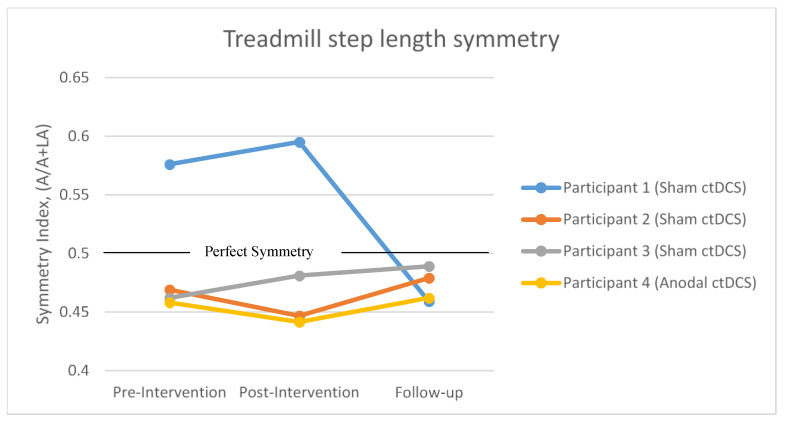
Mean treadmill step length symmetry at pre-intervention, post-intervention and follow-up assessment. The black horizontal line represents perfect symmetry. A: affected step length, LA: less-affected step length.

Refer to Figure 5 for illustration of results. The change in mean treadmill step length symmetry from pre-intervention to post-intervention remained unchanged for participants 1 (sham ctDCS: −0.02), 2 (sham ctDCS: 0.02), 3 (sham ctDCS: 22120.02) and 4 (anodal ctDCS: 0.02).

##### Pre-Intervention to Follow-Up Assessment

The change in mean treadmill step length symmetry from pre-intervention to follow-up remained unchanged for participants 2 (sham ctDCS: −0.01), 3 (sham ctDCS: −0.03) and 4 (anodal ctDCS: −0.004) (Figure 5). However, for participant 1 (sham ctDCS: 0.12), step length symmetry moved closer to the perfect symmetry value of 0.5; this change exceeded the MDC (0.068).

#### 3.3.2. Over-Ground Step Length Symmetry

##### Pre- to Post-Intervention Assessment

**Figure 6:**
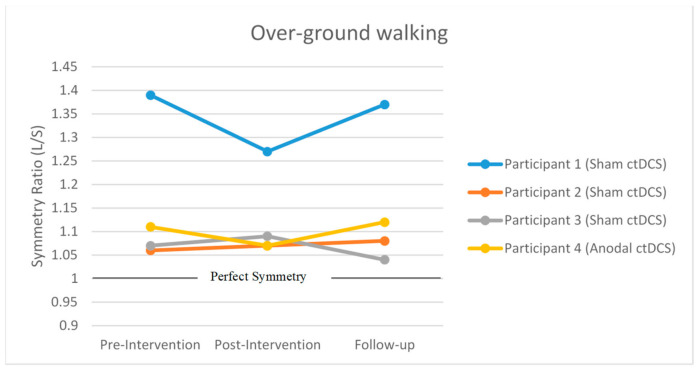
Mean over-ground step length at pre-intervention, post-intervention and follow-up assessment session. The black horizontal line represents perfect symmetry. L: longer step length, S: shorter step length.

The change in mean over-ground step length symmetry from pre-intervention to post-intervention remained unchanged for participants 2 (sham ctDCS: 0.01), 3 (sham ctDCS: −0.02) and 4 (anodal ctDCS: 0.04) (Figure 6). For participant 1 (sham ctDCS: 0.12), the over-ground step length symmetry moved towards the perfect symmetry value of 1 but did not exceed the MDC (0.15).

##### Pre- to Follow-Up Assessment

The change in mean over-ground step length symmetry from pre-intervention to follow-up remained unchanged for participants 1 (sham ctDCS: 0.02), 2 (sham ctDCS: −0.02), 3 (sham ctDCS: 0.03) and 4 (anodal ctDCS: −0.01) (Figure 6).

#### 3.3.3. Comfortable Over-Ground Walking Speed

**Figure 7:**
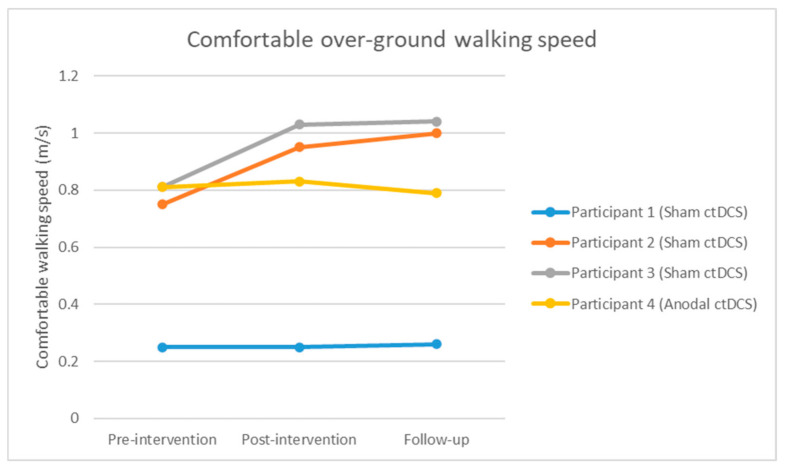
Comfortable walking speed at pre-intervention, post-intervention and follow-up assessment session.

Comfortable over-ground walking speed increased from pre-intervention to post-intervention for participants 2 (0.20 m/s change) and 3 (0.22 m/s change). This improvement in walking speed was maintained at follow-up and exceeded the minimally clinical important difference (MCID) of 0.14m/s [83] (participant 2 sham: 0.25 m/s, participant 3 sham: 0.23 m/s). Comfortable over-ground walking speed remained largely unchanged for participants 1 and 4. Refer to Figure 7.

## 4. Discussion

This is the first study to examine the feasibility of a research protocol investigating three consecutive sessions of anodal ctDCS during SBTT in people with chronic stroke. Determining feasibility is an important prerequisite to evaluating intervention efficacy; this allows the research protocol to be refined and increases the likelihood of implementing a successful level one RCT [84,85]. The intent of the full RCT was to evaluate the effect of repeated anodal ctDCS on motor learning in people with chronic stroke by measuring changes in motor performance in response to locomotor adaptation training. This training paradigm is commonly used in motor learning research [15] and is advocated as a treatment intervention to correct walking asymmetry in people with stroke [16,45]. The findings of this study revealed that the planned RCT protocol and ctDCS-SBTT intervention are not feasible. The main feasibility issues related to the assumptions are that (a) the majority of people with stroke have a walking asymmetry in which the less affected leg has the shorter step length [35,36,37,38] and (b) that this asymmetry can be assessed with video observation [40]. Our data challenged these assumptions. Of the eight people who underwent the lab-based screening, four were excluded as their affected leg had the shorter step length. This illustrates the heterogeneity in step length asymmetry that exists in the stroke population [86], which can be attributed to the diversity in clinical presentation [36]. It suggests that SBTT protocols should not require that the less affected side have the shorter step length; rather, that the leg with the shorter step length, whichever side, be placed on the fast belt. This would improve the recruitment feasibility, enhance the external validity of the research findings, and translation to clinical practice.

In relation to the assumption that walking asymmetry can be assessed with video observation [87,88,89], our study raised several factors that contest this idea. Of the four included participants, there was intra-individual variability in the side with the shorter step length both within the over-ground walking condition and between the over-ground and treadmill walking conditions. This meant that determining which leg to place on the fast treadmill belt was challenging (refer to Table 3). Variability in walking patterns between over-ground and treadmill conditions has been observed in other studies of people with chronic stroke. It has been noted that treadmill walking is associated with shorter stride lengths, faster cadence, and greater step time, stance time and stance-swing time ratios than over-ground walking [74,90]. For one participant in our study, the 3D motion data showed a complete reversal of the side with the shorter step length between over-ground and treadmill walking. Although the exact reason for this is unclear, the use of handrails during treadmill walking may contribute to these differences [91,92,93,94]. Another reason may be that the individuals may switch their strategy to standing as little as possible on the more-affected leg because they are more worried about dynamic balance on the treadmill than are about step length [95]. Given these feasibility issues, future research should determine the validity and reliability of step length asymmetry measurement methods and their relevance to both over-ground and treadmill walking in people with stroke.

In addition to the variability between over-ground and treadmill walking conditions, there were also discrepancies between the video observation and 3D measurements. Post-hoc analysis revealed inconsistencies in step length asymmetry measured using video observation and the 3D motion analysis during treadmill walking in three out of four participants. Whilst, video observation of walking has moderate reliability and validity in people with stroke [87,88], and is time-efficient and cost-effective [96,97,98], it is considered inferior to 3D motion analysis. The reliability of video analysis for determining step length asymmetry is reduced in the absence of marked asymmetry [89]; this may have contributed to the inconsistencies between video and 3D analysis in this study, as most participants had over-ground asymmetry values that did not exceed the asymmetry threshold of a 5cm difference between affected and less-affected legs [45]. Thus, in our study, it appears that the use of video observation contributed to an inaccurate assessment of step length asymmetry, and this meant that for three of the four participants, the leg with the longer step length was placed on the fast belt. This is a deviation from the SBTT intervention recommended in the literature, which states that the initial asymmetry must be exaggerated by placing the leg with the shorter step length on the fast belt [33]. However, there is also evidence for placing the less-affected leg on the fast belt in case the magnitude of baseline step length asymmetry is within the normal symmetry threshold [40]. The assessment of step length asymmetry proved challenging and compromised the fidelity of the SBTT intervention. This is a significant issue for further research. Although 3D analysis may be preferred for its accuracy, it is time-consuming and generally not available in a clinical setting. Therefore, further work is needed to identify a quick and reliable method for determining step length asymmetry if SBTT is to translate into clinical practice.

In addition to intra-individual variability in the side of the shorter step length, there were also differences in the magnitude of step length asymmetry between the over-ground and treadmill walking conditions. An asymmetry threshold represents the cut-off value for the presence or absence of walking asymmetry. Several criteria to determine the asymmetry threshold have been reported in the literature, including the use of an arbitrary value of 10% deviation from perfect symmetry [99], 95% confidence intervals [22,38], or 2 SD [41] of gait symmetry obtained in healthy control participants. The majority of participants, whose asymmetry did not exceed the 5 cm asymmetry threshold during over-ground walking [45], did exceed the 2 cm threshold during treadmill walking [41]. Future studies should consider screening potential participants on the basis of the magnitude of baseline asymmetry, such that only those with marked asymmetry are included. It is also important to consider whether the aim of the intervention is to improve symmetry during over-ground or treadmill walking when choosing over-ground or treadmill baseline asymmetry for screening.

The criterion for recruitment feasibility was that 30 participants would be recruited in four months. This criterion was not met, as only four out of the 26 potential participants were enrolled in the study. In addition to the exclusion of people with a shorter step length on the affected side, other factors that limited recruitment was the presence of contraindications to the use of ctDCS and fear of walking on the split-belt treadmill. With regard to the other criteria for feasibility, retention and data completeness were satisfactory, as well as adherence and safety of the SBTT intervention. The fidelity, adherence, and safety of anodal ctDCS were sufficient; however, this finding must be approached with some caution, as it is inferred from only one participant.

All participant’s motor learning outcomes remained unchanged, except for one participant in the sham ctDCS group who had marked baseline asymmetry and received SBTT with the leg with the shorter step length on the fast belt (as per protocol). This participant had more symmetrical step lengths at follow-up, indicating that SBTT alone had resulted in the retention of this improved walking pattern (i.e., motor learning had occurred). This change exceeded the threshold for MDC. A clinically meaningful improvement being evident in one participant and not others likely highlights the importance of correctly determining the magnitude and side of baseline step length asymmetry, as this participant’s asymmetry exceeded the 5cm threshold and the leg with the shorter step length was correctly allocated to the fast belt during SBTT. Therefore, to maximize the efficacy of the intervention, it is necessary to ensure people with stroke have a magnitude of asymmetry which will respond to SBTT and that the appropriate belt speeds are used during SBTT.

It is also noteworthy that improvements in treadmill walking for this participant did not transfer to over-ground walking. This finding is contrary to previous studies, which have found improvement in over-ground step length symmetry following repeated SBTT alone [16,45]. However, these improvements have been reported following higher doses of SBTT and using over-ground step length symmetry to allocate belt speeds during SBTT. Another explanation could be related to the way the errors were introduced. In both healthy and people with chronic stroke, greater transfer to over-ground walking is noted when the belt speed is changed slowly but not abruptly [100,101]. The slow change in belt speeds induces smaller errors, which may fall within the individual’s baseline variability such that one adapts to natural over-ground walking patterns. In contrast, an abrupt change in belt speed produces large errors beyond the normal range resulting in an adapted pattern that does not transfer, regardless of the gains in motor learning over the treadmill [100]. Furthermore, individuals may respond to abrupt perturbations explicitly, while they may respond to gradual perturbations implicitly. Implicit adaptation is thought to lead to the development of an internal model, whereas explicit adaptation does not [102]. Based on the pattern of data observed from participant 1 (see Figure 4), it seems likely that this participant dealt with the perturbation explicitly (i.e., cognitively). This may explain the lack of transfer from one setting to another. Therefore, in our study, the use of abrupt change in the belt speed may have resulted in the lack of transfer to over-ground walking. Overall, factors such as SBTT dose, type and size of error may influence transfer to over-ground walking. Therefore, these factors need to be considered when designing future studies.

In all the participants, improvements in comfortable over-ground walking speed had no relation to whether the symmetry improved or remained unchanged. In participants receiving sham ctDCS, over-ground comfortable walking speed did not change for the participant who displayed improved symmetry. Two sham participants, who did not have improved step symmetry, experienced improvements in walking speed that exceeded the minimal clinically important difference at both post-intervention and follow-up assessment. This improved walking speed may be related to other compensatory mechanisms adopted by the participants during the intervention [99]. However, data from more participants are required to determine the effect of SBTT on walking speed.

### Limitations, Implications and Future Research

One of the main limitations of this study was the lack of qualitative data to determine the acceptability of the intervention. Considering the findings of other feasibility measures, it is unlikely that this would have altered the main findings of the study. Another limitation was that the included participants could not be described based on their lesion location as they were recruited through the community. Inferring the feasibility of anodal ctDCS from a single participant who received the intervention was also a limitation. The short duration of ctDCS stimulation is another limitation as it may be responsible for the lack of ctDCS effects in the participant who received anodal ctDCS. Future stroke studies need to optimize the stimulation duration with respect to SBTT adaptation bouts. Lastly, the inclusion of a three-minute de-adaptation phase may have caused some washout of adapted patterns. Despite these limitations, the study identified a number of unanticipated issues that highlight the importance of evaluating the feasibility of an intervention and research protocol prior to a larger trial. These issues, primarily relating to the variability in step length asymmetry, can be overcome by recruiting stroke participants with shorter step length on either side, including stroke participants who have baseline asymmetry above the normal asymmetry threshold, and setting up SBTT with respect to the individual’s step length asymmetry. Given the lack of clarity in SBTT research with regard to how and to whom SBTT is best delivered for people with stroke, alternative methods of evaluating motor learning during adaptation in people with stroke should be considered. The efficacy of repeated ctDCS in people with stroke may be more appropriately investigated using motor adaptation tasks such as force-field tasks applying robot-induced forces to upper limb reaching movements [103], or locomotor tasks involving unilateral leg weighting during treadmill walking [104,105] or spatio-temporal cues during over-ground walking [106].

## 5. Conclusions

The planned RCT research protocol constituting three consecutive sessions of intervention is not feasible in its current form. The study revealed substantial variability in the direction of step length asymmetry influencing the recruitment and delivery of SBTT. This highlights the challenges of delivering an intervention which relies on the assessment of highly variable baseline measures to assure successful error augmentation. The efficacy of ctDCS to influence motor re-learning during motor adaptation in people with stroke is still not known. Future studies need to either resolve feasibility issues around the identification of step length asymmetry and the assignment of it during SBTT or utilize an alternative motor adaptation paradigm to determine the effects of repeated ctDCS on motor learning in people with stroke.

## Figures and Tables

**Table 1:** Feasibility criteria and assessment.

Feasibility Criteria	Questions Asked	Records Of
Recruitment	Can 30 participants be recruited for the study within four months?	Number of participants considered, screened, and included Reasons for exclusion
Retention	Is the drop-out rate of participants not more than 20%?	Number of participants who dropped out of the trial Reason for dropping out
Protocol Deviation	Can the deviations in the protocol be addressed with minor alterations to the protocol and its implementation?	Any deviations from the described protocol
Data Completeness	Does data completion exceed 95%?	Missing data Reasons for missing
Intervention Adherence	Does the participant’s adherence to the intervention exceed 80%?	Session attendance
Intervention Fidelity	Can the anodal ctDCS be delivered as per the planned protocol such that the fidelity exceeds 80%?	Stimulation location Stimulation intensity Stimulation duration
	Can the SBTT be delivered as per the planned protocol such that the fidelity exceeds 70%?	Setup of fast and slow belt speed Allocation of limbs to split-belt condition Duration of each phase and rest break
Intervention Safety	Is the three consecutive days of anodal ctDCS safe?	Medical or physical changes at the beginning of each session and during the session Any adverse events reported Description and rating of participant’s experience with the ctDCS stimulation
	Is the three consecutive days of SBTT safe?	Medical or physical changes at the beginning of each session and during the session Any adverse events reported

**Table 2:** Demographics and stroke characteristics.

P.N >	1	2	3	4
Sex	F	F	M	M
Age (yrs)	64	74	51	80
Lesion Type	I	H	H	I
Ethnicity	NZ European	NZ European	Maori Chinese	NZ
Affected Side	L	R	L	R
Time Since Stroke (Months)	240	12	171	62
OG Walking Speed (m/s)	0.25	0.75	0.81	0.81
TM Walking Speed (m/s)	0.20	0.60	0.65	0.65
NIHSS Score	2	0	0	1
SMRS Score	2	2	1	1
Gait Aid	cane	nil	nil	cane
Group	S	S	S	A

P.N: participant number, F: female, M: male, I: ischemic, H: hemorrhagic OG: over-ground, TM: treadmill, NIHSS: National Institute of Health Stroke Scale, SMRS: simplified modified Rankin Scale, S: sham ctDCS, A: active anodal ctDCS.

**Table 3:** Comparison of step length asymmetry direction and step length difference.

P.N.	Step Length Symmetry	Video Observation	3D Motion Data			
			Mean Step Length	Difference in Step Lengths, cm (Mean ± SD)	Exceeds Asymmetry Threshold	Leg with Shorter Step Length Placed on Fast Belt (Fidelity)
1	Over-ground	V	L > A	9.30 ± 3.70	✓ *	✓
	Treadmill	A > L	A > L	6.21 ± 2.45	✓ ^	✓
2	Over-ground	V	L > A	1.30 ± 2.75	X *	✓
	Treadmill	A > L	L > A	5.71 ± 4.55	✓ ^	X
3	Over-ground	V	L > A	1.99 ± 3.58	X *	✓
	Treadmill	A > L	L > A	8.36 ± 3.55	✓ ^	X
4	Over-ground	V	L > A	2.2 ± 6.09	X *	✓
	Treadmill	A > L	L > A	8.01 ± 3.00	✓ ^	X

V: variability in the side with shorter step length, A: affected leg, L: less-affected leg, *: asymmetry threshold 5 cm determined from Reisman et al. 2013 [45], ^: asymmetry threshold 2cm determined from Reisman et al. 2007 [41].

## Data Availability

The datasets analyzed during the current study are available from the corresponding author on reasonable request.

## Abbreviations

3D	3 Dimensional
10MWT	Timed 10-metre walk test
ctDCS	Cerebellar transcranial direct current stimulation
Hz	Hertz
mA	Milli Ampere
MCID	Minimally Clinical Important Difference
MDC	Minimal Detectable Change
NIHSS	National Institute of Health Stroke Scale
RCT	Randomized Controlled Trial
SBTT	Split-Belt Treadmill Training
SMRS	Simplified Modified Rankin Scale
STS	Strides To Steady-State
tDCS	Transcranial direct current stimulation

## References

[1] Feigin V.L., Abajobir A.A., Abate K.H., Abd-Allah F., Abdulle A.M., Abera S.F., Abyu G.Y., Ahmed M.B., Aichour A.N., Aichour I. (2017). Global, regional, and national burden of neurological disorders during 1990–2015: A systematic analysis for the Global Burden of Disease Study 2015. Lancet Neurol..

[2] Cramer S.C. (2008). Repairing the human brain after stroke: I. Mechanisms of spontaneous recovery. Ann. Neurol..

[3] Johansson B.B. (2000). Brain plasticity and stroke rehabilitation: The Willis lecture. Stroke.

[4] Legg L., Langhorne P., Drummond A., Gladman J., Logan P., Walker M. (2004). Rehabilitation therapy services for stroke patients living at home: Systematic review of randomised controlled trials. Lancet.

[5] Van Peppen R.P., Kwakkel G., Wood-Dauphinee S., Hendriks H.J., Van der Wees P.J., Dekker J. (2004). The impact of physical therapy on functional outcomes after stroke: What’s the evidence?. Clin. Rehabil..

[6] Gresham G.E., Fitzpatrick T.E., Wolf P.A., McNamara P.M., Kannel W.B., Dawber T.R. (1975). Residual disability in survivors of stroke—The Framingham study. N. Engl. J. Med..

[7] Langhorne P., Bernhardt J., Kwakkel G. (2011). Stroke rehabilitation. Lancet.

[8] Fulk G.D., He Y., Boyne P., Dunning K. (2017). Predicting home and community walking activity poststroke. Stroke.

[9] Woodman P., Riazi A., Pereira C., Jones F. (2014). Social participation post stroke: A meta-ethnographic review of the experiences and views of community-dwelling stroke survivors. Disabil. Rehabil..

[10] Krakauer J.W. (2006). Motor learning: Its relevance to stroke recovery and neurorehabilitation. Curr. Opin. Neurol..

[11] Dimyan M.A., Cohen L.G. (2011). Neuroplasticity in the context of motor rehabilitation after stroke. Nat. Rev. Neurol..

[12] Schmidt R.A., Lee T.D. (2011). Motor Control and Learning: A Behavioral Emphasis.

[13] Martin T.A., G Keating J., P Goodkin H., Bastian A.J., Thach W. (1996). Throwing while looking through prisms. II. Specificity and storage of multiple gaze-throw calibrations. Brain.

[14] Bastian A.J. (2008). Understanding sensorimotor adaptation and learning for rehabilitation. Curr. Opin. Neurol..

[15] Reisman D.S., Bastian A.J., Morton S.M. (2010). Neurophysiologic and Rehabilitation Insights From the Split-Belt and Other Locomotor Adaptation Paradigms. Phys. Ther..

[16] Betschart M., McFadyen B.J., Nadeau S. (2018). Repeated split-belt treadmill walking improved gait ability in individuals with chronic stroke: A pilot study. Physiother. Theory Pract..

[17] Awad L.N., Palmer J.A., Pohlig R.T., Binder-Macleod S.A., Reisman D.S. (2015). Walking speed and step length asymmetry modify the energy cost of walking after stroke. Neurorehabil. Neural Repair.

[18] Sánchez N., Simha S.N., Donelan J.M., Finley J.M. (2019). Taking advantage of external mechanical work to reduce metabolic cost: The mechanics and energetics of split-belt treadmill walking. J. Physiol..

[19] Hendrickson J., Patterson K.K., Inness E.L., McIlroy W.E., Mansfield A. (2014). Relationship between asymmetry of quiet standing balance control and walking post-stroke. Gait Posture.

[20] Lewek M.D., Bradley C.E., Wutzke C.J., Zinder S.M. (2014). The relationship between spatiotemporal gait asymmetry and balance in individuals with chronic stroke. J. Appl. Biomech..

[21] Jørgensen L., Crabtree N., Reeve J., Jacobsen B. (2000). Ambulatory level and asymmetrical weight bearing after stroke affects bone loss in the upper and lower part of the femoral neck differently: Bone adaptation after decreased mechanical loading. Bone.

[22] Patterson K.K., Parafianowicz I., Danells C.J., Closson V., Verrier M.C., Staines W.R., Black S.E., McIlroy W.E. (2008). Gait asymmetry in community-ambulating stroke survivors. Arch. Phys. Med. Rehabil..

[23] Hesse S., Werner C., Bardeleben A., Barbeau H. (2001). Body weight-supported treadmill training after stroke. Curr. Atheroscler. Rep..

[24] Titianova E.B., Tarkka I.M. (1995). Asymmetry in walking performance and postural sway in patients with chronic unilateral cerebral infarction. J. Rehabil. Res. Dev..

[25] Regnaux J., Pradon D., Roche N., Robertson J., Bussel B., Dobkin B. (2008). Effects of loading the unaffected limb for one session of locomotor training on laboratory measures of gait in stroke. Clin. Biomech..

[26] Patterson S.L., Rodgers M.M., Macko R.F., Forrester L.W. (2008). Effect of treadmill exercise training on spatial and temporal gait parameters in subjects with chronic stroke: A preliminary report. J. Rehabil. Res. Dev..

[27] Silver K.H., Macko R.F., Forrester L.W., Goldberg A.P., Smith G.V. (2000). Effects of aerobic treadmill training on gait velocity, cadence, and gait symmetry in chronic hemiparetic stroke: A preliminary report. Neurorehabil. Neural Repair.

[28] Patterson K.K., Mansfield A., Biasin L., Brunton K., Inness E.L., McIlroy W.E. (2015). Longitudinal changes in poststroke spatiotemporal gait asymmetry over inpatient rehabilitation. Neurorehabil. Neural Repair.

[29] Combs S.A., Dugan E.L., Ozimek E.N., Curtis A.B. (2013). Bilateral coordination and gait symmetry after body-weight supported treadmill training for persons with chronic stroke. Clin. Biomech..

[30] Lamontagne A., Fung J. (2004). Faster is better: Implications for speed-intensive gait training after stroke. Stroke.

[31] Thaut M., Leins A., Rice R., Argstatter H., Kenyon G., McIntosh G., Bolay H., Fetter M. (2007). Rhythmic auditor y stimulation improves gait more than NDT/Bobath training in near-ambulatory patients early poststroke: A single-blind, randomized trial. Neurorehabil. Neural Repair.

[32] Reisman D.S., Block H.J., Bastian A.J. (2005). Interlimb coordination during locomotion: What can be adapted and stored?. J. Neurophysiol..

[33] Tyrell C.M., Helm E., Reisman D.S. (2015). Locomotor adaptation is influenced by the interaction between perturbation and baseline asymmetry after stroke. J. Biomech..

[34] Jayaram G., Tang B., Pallegadda R., Vasudevan E.V., Celnik P., Bastian A. (2012). Modulating locomotor adaptation with cerebellar stimulation. J. Neurophysiol..

[35] Hsu A.-L., Tang P.-F., Jan M.-H. (2003). Analysis of impairments influencing gait velocity and asymmetry of hemiplegic patients after mild to moderate stroke. Arch. Phys. Med. Rehabil..

[36] Balasubramanian C.K., Neptune R.R., Kautz S.A. (2009). Variability in spatiotemporal step characteristics and its relationship to walking performance post-stroke. Gait Posture.

[37] Lauziere S., Betschart M., Aissaoui R., Nadeau S. (2014). Understanding spatial and temporal gait asymmetries in individuals post stroke. Int. J. Phys. Med. Rehabil..

[38] Patterson K.K., Gage W.H., Brooks D., Black S.E., McIlroy W.E. (2010). Evaluation of gait symmetry after stroke: A comparison of current methods and recommendations for standardization. Gait Posture.

[39] Malone L.A., Bastian A.J. (2014). Spatial and temporal asymmetries in gait predict split-belt adaptation behavior in stroke. Neurorehabil. Neural Repair.

[40] Reisman D.S., Wityk R., Silver K., Bastian A.J. (2009). Split-belt treadmill adaptation transfers to overground walking in persons poststroke. Neurorehabil. Neural Repair.

[41] Reisman D.S., Wityk R., Silver K., Bastian A.J., Reisman D.S., Wityk R., Silver K., Bastian A.J. (2007). Locomotor adaptation on a split-belt treadmill can improve walking symmetry post-stroke. Brain A J. Neurol..

[42] Savin D.N., Tseng S.-C., Whitall J., Morton S.M. (2013). Poststroke hemiparesis impairs the rate but not magnitude of adaptation of spatial and temporal locomotor features. Neurorehabil. Neural Repair.

[43] Tyrell C.M., Helm E., Reisman D.S. (2014). Learning the spatial features of a locomotor task is slowed after stroke. J. Neurophysiol..

[44] Reisman D.S., McLean H., Bastian A.J. (2010). Split-belt treadmill training poststroke: A case study. J. Neurol. Phys. Ther..

[45] Reisman D.S., McLean H., Keller J., Danks K.A., Bastian A.J. (2013). Repeated split-belt treadmill training improves poststroke step length asymmetry. Neurorehabil. Neural Repair.

[46] Di Pino G., Pellegrino G., Assenza G., Capone F., Ferreri F., Formica D., Ranieri F., Tombini M., Ziemann U., Rothwell J.C. (2014). Modulation of brain plasticity in stroke: A novel model for neurorehabilitation. Nat. Rev. Neurol..

[47] Madhavan S. (2017). Magnetic and Direct Current Stimulation for Stroke. Primer on Cerebrovascular Diseases.

[48] Nitsche M.A., Paulus W. (2000). Excitability changes induced in the human motor cortex by weak transcranial direct current stimulation. J. Physiol..

[49] Nitsche M.A., Paulus W. (2011). Transcranial direct current stimulation—Update 2011. Restor. Neurol. Neurosci..

[50] Awosika O.O., Cohen L.G., Knotkova H., Nitsche M.A., Bikson M., Woods A.J. (2019). Transcranial Direct Current Stimulation in Stroke Rehabilitation: Present and Future. Practical Guide to Transcranial Direct Current Stimulation: Principles, Procedures and Applications.

[51] Kindred J.H., Kautz S.A., Wonsetler E.C., Bowden M.G. (2019). Single sessions of high-definition Transcranial direct current stimulation Do not Alter lower extremity biomechanical or Corticomotor response variables post-stroke. Front. Neurosci..

[52] Celnik P. (2015). Understanding and modulating motor learning with cerebellar stimulation. Cerebellum.

[53] Jayaram G., Galea J.M., Bastian A.J., Celnik P. (2011). Human locomotor adaptive learning is proportional to depression of cerebellar excitability. Cereb. Cortex.

[54] Block H.J., Celnik P. (2012). Can cerebellar transcranial direct current stimulation become a valuable neurorehabilitation intervention?. Expert Rev. Neurother..

[55] van Dun K., Bodranghien F., Marien P., Manto M.U. (2016). tDCS of the cerebellum: Where do we stand in 2016? technical issues and critical review of the literature. Front. Hum. Neurosci..

[56] Kumari N., Taylor D., Signal N. (2019). The effect of cerebellar transcranial direct current stimulation on motor learning: A systematic review of randomized controlled trials. Front. Hum. Neurosci..

[57] Cantarero G., Spampinato D., Reis J., Ajagbe L., Thompson T., Kulkarni K., Celnik P. (2015). Cerebellar direct current stimulation enhances on-line motor skill acquisition through an effect on accuracy. J. Neurosci..

[58] Kumari N., Taylor D., Rashid U., Vandal A.C., Smith P.F., Signal N. (2020). Cerebellar transcranial direct current stimulation for learning a novel split-belt treadmill task: A randomised controlled trial. Sci. Rep..

[59] Zandvliet S.B., Meskers C.G.M., Kwakkel G., van Wegen E.E.H. (2018). Short-Term Effects of Cerebellar tDCS on Standing Balance Performance in Patients with Chronic Stroke and Healthy Age-Matched Elderly. Cerebellum.

[60] Thair H., Holloway A.L., Newport R., Smith A.D. (2017). Transcranial direct current stimulation (tDCS): A beginner’s guide for design and implementation. Front. Neurosci..

[61] Helm E.E., Tyrell C.M., Pohlig R.T., Brady L.D., Reisman D.S. (2016). The presence of a single-nucleotide polymorphism in the BDNF gene affects the rate of locomotor adaptation after stroke. Exp. Brain Res..

[62] Lewek M.D., Braun C.H., Wutzke C., Giuliani C. (2018). The role of movement errors in modifying spatiotemporal gait asymmetry post stroke: A randomized controlled trial. Clin. Rehabil..

[63] DeLisa J.A. (1998). Gait Analysis in the Science of Rehabilitation.

[64] Bassile C.C., Hayes S.M. (2016). Gait awareness. Stroke Rehabilitation.

[65] Evans S., Royston P., Day S. (2004). Minim: Allocation by Minimisation in Clinical Trials. https://www.semanticscholar.org/paper/Minim%3A-allocation-by-minimisation-in-clinical-Evans-Royston/e9dce94277e10390136caeb0e1f61c35815da0d0.

[66] Taves D.R. (2010). The use of minimization in clinical trials. Contemp. Clin. Trials.

[67] Schulz K.F., Altman D.G., Moher D. (2010). CONSORT 2010 statement: Updated guidelines for reporting parallel group randomised trials. BMC Med..

[68] Ortiz G.A., Sacco R.L. (2014). National institutes of health stroke scale (nihss). Wiley Statsref Stat. Ref. Online.

[69] Bruno A., Close B., Switzer J.A., Hess D.C., Gross H., Nichols III F.T., Akinwuntan A.E. (2013). Simplified modified Rankin Scale questionnaire correlates with stroke severity. Clin. Rehabil..

[70] Betschart M., Lauzière S., Miéville C., McFadyen B.J., Nadeau S. (2017). Changes in lower limb muscle activity after walking on a split-belt treadmill in individuals post-stroke. J. Electromyogr. Kinesiol..

[71] Sutherland D.H. (2002). The evolution of clinical gait analysis: Part II Kinematics. Gait Posture.

[72] Rashid U., Kumari N., Taylor D., David T., Signal N. (2019). Gait event anomaly detection and correction during a split-belt treadmill task. IEEE Access.

[73] Vasudevan E.V., Bastian A.J. (2009). Split-belt treadmill adaptation shows different functional networks for fast and slow human walking. J. Neurophysiol..

[74] Bayat R., Barbeau H., Lamontagne A. (2005). Speed and temporal-distance adaptations during treadmill and overground walking following stroke. Neurorehabil. Neural Repair.

[75] Schlerf J.E., Galea J.M., Spampinato D., Celnik P.A. (2014). Laterality differences in cerebellar–motor cortex connectivity. Cereb. Cortex.

[76] Herzfeld D.J., Pastor D., Haith A.M., Rossetti Y., Shadmehr R., O’Shea J. (2014). Contributions of the cerebellum and the motor cortex to acquisition and retention of motor memories. NeuroImage.

[77] Kesar T.M., Binder-Macleod S.A., Hicks G.E., Reisman D.S. (2011). Minimal detectable change for gait variables collected during treadmill walking in individuals post-stroke. Gait Posture.

[78] Lewek M.D., Randall E.P. (2011). Reliability of spatiotemporal asymmetry during overground walking for individuals following chronic stroke. J. Neurol. Phys. Ther..

[79] Patterson K.K., Gage W.H., Brooks D., Black S.E., McIlroy W.E. (2010). Changes in gait symmetry and velocity after stroke: A cross-sectional study from weeks to years after stroke. Neurorehabil. Neural Repair.

[80] Flansbjer U.-B., Holmbäck A.M., Downham D., Patten C., Lexell J. (2005). Reliability of gait performance tests in men and women with hemiparesis after stroke. J. Rehabil. Med..

[81] Vos-Vromans D., Ketelaar M., Gorter J. (2005). Responsiveness of evaluative measures for children with cerebral palsy: The Gross Motor Function Measure and the Pediatric Evaluation of Disability Inventory. Disabil. Rehabil..

[82] Perry J., Garrett M., Gronley J.K., Mulroy S.J. (1995). Classification of walking handicap in the stroke population. Stroke.

[83] Perera S., Mody S.H., Woodman R.C., Studenski S.A. (2006). Meaningful change and responsiveness in common physical performance measures in older adults. J. Am. Geriatr. Soc..

[84] Orsmond G.I., Cohn E.S. (2015). The Distinctive Features of a Feasibility Study: Objectives and Guiding Questions. OTJR.

[85] Tickle-Degnen L. (2013). Nuts and bolts of conducting feasibility studies. Am. J. Occup. Ther..

[86] Chen G., Patten C., Kothari D.H., Zajac F.E. (2005). Gait differences between individuals with post-stroke hemiparesis and non-disabled controls at matched speeds. Gait Posture.

[87] Lord S., Halligan P., Wade D. (1998). Visual gait analysis: The development of a clinical assessment and scale. Clin. Rehabil..

[88] Miyazaki S., Kubota T. (1984). Quantification of gait abnormalities on the basis of continuous foot-force measurement: Correlation between quantitative indices and visual rating. Med. Biol. Eng. Comput..

[89] Hughes K.A., Bell F. (1994). Visual assessment of hemiplegic gait following stroke: Pilot study. Arch. Phys. Med. Rehabil..

[90] Puh U., Baer G.D. (2009). A comparison of treadmill walking and overground walking in independently ambulant stroke patients: A pilot study. Disabil. Rehabil..

[91] Harris-Love M.L., Forrester L.W., Macko R.F., Silver K.H., Smith G.V. (2001). Hemiparetic gait parameters in overground versus treadmill walking. Neurorehabil. Neural Repair.

[92] Kuys S.S., Brauer S.G., Ada L., Russell T.G. (2008). Increasing intensity during treadmill walking does not adversely affect walking pattern or quality in newly-ambulating stroke patients: An experimental study. Aust. J. Physiother..

[93] Chen G., Patten C., Kothari D.H., Zajac F.E. (2005). Gait deviations associated with post-stroke hemiparesis: Improvement during treadmill walking using weight support, speed, support stiffness, and handrail hold. Gait Posture.

[94] Chen G., Patten C. (2006). Treadmill training with harness support: Selection of parameters for individuals with poststroke hemiparesis. J. Rehabil. Res. Dev..

[95] Darter B.J., Labrecque B.A., Perera R.A. (2018). Dynamic stability during split-belt walking and the relationship with step length symmetry. Gait Posture.

[96] Coutts F. (1999). Gait analysis in the therapeutic environment. Man. Ther..

[97] Malouin F. (1995). Gait Analysis: Theory and Application.

[98] Harris G.F., Wertsch J.J. (1994). Procedures for gait analysis. Arch. Phys. Med. Rehabil..

[99] Balasubramanian C.K., Bowden M.G., Neptune R.R., Kautz S.A. (2007). Relationship between step length asymmetry and walking performance in subjects with chronic hemiparesis. Arch. Phys. Med. Rehabil..

[100] Alcântara C.C., Charalambous C.C., Morton S.M., Russo T.L., Reisman D.S. (2018). Different Error Size During Locomotor Adaptation Affects Transfer to Overground Walking Poststroke. Neurorehabil. Neural Repair.

[101] Torres-Oviedo G., Bastian A.J. (2011). Natural error patterns enable transfer of motor learning to novel contexts. J. Neurophysiol..

[102] Wang J., Bao S., Tays G.D. (2019). Lack of generalization between explicit and implicit visuomotor learning. PLoS ONE.

[103] Patton J.L., Mussa-Ivaldi F.A. (2004). Robot-assisted adaptive training: Custom force fields for teaching movement patterns. IEEE Trans. Biomed. Eng..

[104] Damiano D.L., Stanley C.J., Bulea T.C., Park H.S. (2017). Motor learning abilities are similar in hemiplegic cerebral palsy compared to controls as assessed by adaptation to unilateral leg-weighting during gait: Part I. Front. Hum. Neurosci..

[105] Noble J.W., Prentice S.D. (2006). Adaptation to unilateral change in lower limb mechanical properties during human walking. Exp. Brain Res..

[106] Fernandez L., Albein-Urios N., Kirkovski M., McGinley J.L., Murphy A.T., Hyde C., Stokes M.A., Rinehart N.J., Enticott P.G. (2017). Cathodal transcranial direct current stimulation (tDCS) to the right cerebellar hemisphere affects motor adaptation during gait. Cerebellum.

